# Molecular dynamics study on characteristics of reflection and condensation molecules at vapor–liquid equilibrium state

**DOI:** 10.1371/journal.pone.0248660

**Published:** 2021-03-16

**Authors:** Hirofumi Tabe, Kazumichi Kobayashi, Hiroyuki Fujii, Masao Watanabe

**Affiliations:** Division of Mechanical and Space Engineering, Hokkaido University, Sapporo, Hokkaido, Japan; Tsinghua University, CHINA

## Abstract

The kinetic boundary condition (KBC) represents the evaporation or condensation of molecules at the vapor–liquid interface for molecular gas dynamics (MGD). When constructing the KBC, it is necessary to classify molecular motions into evaporation, condensation, and reflection in molecular-scale simulation methods. Recently, a method that involves setting the vapor boundary and liquid boundary has been used for classifying molecules. The position of the vapor boundary is related to the position where the KBC is applied in MGD analyses, whereas that of the liquid boundary has not been uniquely determined. Therefore, in this study, we conducted molecular dynamics simulations to discuss the position of the liquid boundary for the construction of KBCs. We obtained some variables that characterize molecular motions such as the positions that the molecules reached and the time they stayed in the vicinity of the interface. Based on the characteristics of the molecules found from these variables, we investigated the valid position of the liquid boundary. We also conducted an investigation on the relationship between the condensation coefficient and the molecular incident velocity from the vapor phase to the liquid phase. The dependence of the condensation coefficient on the incident velocity of molecules was confirmed, and the value of the condensation coefficient becomes small in the low-incident-velocity range. Furthermore, we found that the condensation coefficient in the non-equilibrium state shows almost the same value as that in the equilibrium state, although the corresponding velocity distribution functions of the incident velocity significantly differ from each other.

## 1 Introduction

Gas or vapor flows in the non-equilibrium region in the vicinity of the vapor–liquid interface can be investigated based on molecular gas dynamics (MGD) [1–3], which is described using the velocity distribution function of gas/vapor molecules. In MGD analyses, macroscopic values (density, velocity, temperature, etc.) at an arbitrary position and mass, momentum, and energy fluxes passing through an arbitrary surface in gas/vapor phases can be calculated by solving velocity distribution functions of molecules, which are governed by the Boltzmann equation.

In previous studies, bubble collapse [4, 5], droplet evaporation [6], and nanoporous evaporation [7] have been investigated via MGD analysis taking into consideration the evaporation and condensation of molecules at the interface. When investigating physical phenomena accompanied by evaporation/condensation in MGD analysis, we need to impose a boundary condition on the vapor–liquid interface that represents the evaporation and condensation of molecules. This boundary condition is called the kinetic boundary condition (KBC). Evaporation from the liquid phase and condensation into it cannot be accurately represented without KBCs and, therefore, setting them is indispensable for MGD analysis of phase change phenomena. Because the Boltzmann equation for MGD analyses governs the temporal and spatial evolutions of the velocity distribution function of gas/vapor molecules, the KBC also has the form of the velocity distribution function.

For the vapor molecules outgoing to the vapor phase from the liquid phase, the KBC is given by the following function [8]:

$$\begin{array}{c} f_{\text{out}}=\frac{\alpha_{e}\rho^{*}+\left(1-\alpha_{c}\right)\sigma }{{\left(\sqrt{2\pi RT_{L}}\right)}^{3}}\;\text{exp}(-\frac{\xi_{x}^{2}+\xi_{y}^{2}+\xi_{z}^{2}}{2RT_{L}}),\;\text{for}\;\xi_{z}>0, \end{array}$$ (1)

where *R* is the gas constant of the vapor, *T*_L_ is the liquid temperature, ρ* is the saturated vapor density at *T*_L_, and *σ* is a parameter related to the density of molecules that collide with the vapor–liquid interface from the vapor phase. ***ξ*** = (*ξ*_*x*_, *ξ*_*y*_, *ξ*_*z*_) denotes the molecular velocities along the *x*-, *y*-, and *z*-directions, where *z* is the direction normal to the liquid surface. *σ* is defined by

$$\begin{array}{c} \sigma =-\sqrt{\frac{2\pi }{RT_{L}}}\int_{-\infty }^{0}\int_{-\infty }^{\infty }\int_{-\infty }^{\infty }\xi_{z}f_{\text{coll}}\;d\xi_{x}d\xi_{y}d\xi_{z}, \end{array}$$ (2)

where *f*_coll_ is the velocity distribution function of the molecules having *ξ*_*z*_ < 0 at the vapor–liquid interface and colliding with the interface from the vapor phase. *f*_coll_ can be obtained by solving the Boltzmann equation in MGD analysis.

**Fig 1:**
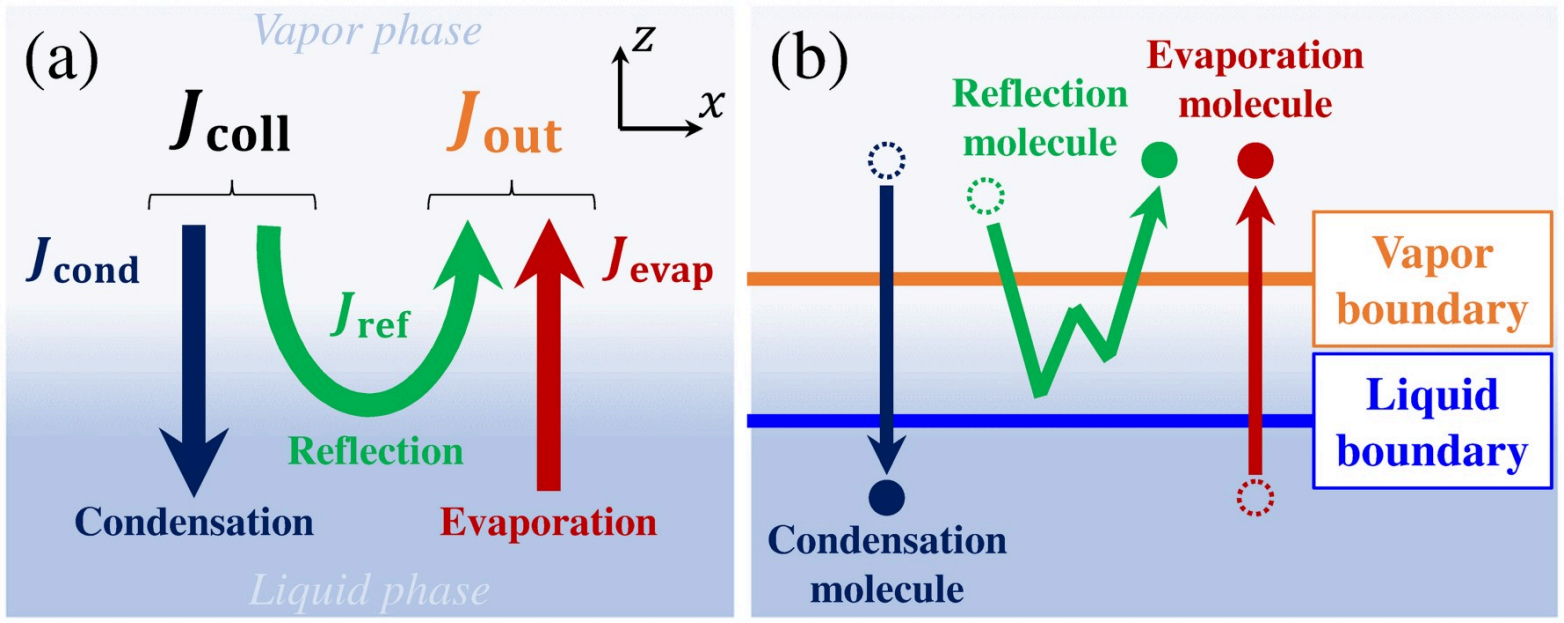
(a) Mass fluxes in the vicinity of vapor–liquid interface; (b) classification of molecules with vapor and liquid boundaries.

*α*_e_ and *α*_c_ in Eq 1 are the evaporation and condensation coefficients, and they have been widely defined as the ratio of mass fluxes of molecules [8–13] as follows:

$$\begin{array}{c} \alpha_{e}=\frac{J_{\text{evap}}}{J_{\text{out}}^{*}},\;\alpha_{c}=\frac{J_{\text{cond}}}{J_{\text{coll}}}, \end{array}$$ (3)

where *J*_evap_ is the mass flux of the evaporation molecules, $J_{\text{out}}^{*}$ is the mass flux of the outgoing molecules from the liquid phase to the vapor phase in the vapor–liquid equilibrium state given by $J_{\text{out}}^{*}=\rho^{*}\sqrt{RT_{L}/2\pi }$, *J*_cond_ is the mass flux of the condensation molecules, and *J*_coll_ is the mass flux of the colliding molecules from the vapor phase to the liquid phase. In addition, there is the other mass flux *J*_ref_ of molecules that are reflected at the vapor–liquid interface and return to the vapor phase. The relationships between these fluxes defined in the vicinity of the vapor–liquid interface are shown in Fig 1a. The mass fluxes of the outgoing molecules, *J*_out_, and the colliding molecules, *J*_coll_, are defined as

$$\begin{array}{c} J_{\text{out}}=J_{\text{evap}}+J_{\text{ref}},\;J_{\text{coll}}=J_{\text{ref}}+J_{\text{cond}}. \end{array}$$ (4)

As shown in the above definitions, *α*_e_ and *α*_c_ indicate the evaporation rate and condensation rate of the molecules, respectively.

When constructing KBCs for MGD analyses, we need to obtain the molecular velocity distributions and mass fluxes as represented in Eqs 1–3. Because these values cannot be obtained from MGD analyses, it is necessary to conduct molecular-scale simulations that can analyze the motions of individual molecules in detail. In particular, the definitions of *α*_e_ and *α*_c_ make it necessary to classify molecules in the vicinity of the vapor–liquid interface into evaporation, reflection, and condensation molecules as represented in Eqs 3 and 4. Currently, several studies using the molecular dynamics (MD) simulation [9, 10, 12–20] or the Enskog–Vlasov direct simulation Monte Carlo (EV-DSMC) method [8, 11, 21–25] have been conducted to investigate evaporation and condensation from the standpoint of molecular motions. However, the classification criterion for molecules is not uniquely defined, because there is no clear definition of evaporation, reflection, and condensation of molecules. Thus, there have been various discussions on the method of classifying molecules, and several approaches to the construction of KBCs have been proposed in previous studies [8, 9, 11–15, 22, 24, 26].

For defining molecular motions and classifying molecules, a method that involves setting two imaginary boundaries, which was proposed by Meland et al. [15] and Gu et al. [26], has been utilized in some previous studies [12, 13, 15, 24, 26]. The relationship between the two imaginary boundaries—the liquid boundary and vapor boundary—and classified molecules is shown in Fig 1b. In this method, a molecule that passes two boundaries from the liquid phase to the vapor phase is defined as an evaporation molecule; a molecule that passes two boundaries from the vapor phase to the liquid phase is defined as a condensation molecule; a molecule that passes the vapor boundary from the vapor phase and returns to the vapor phase without passing the liquid boundary is defined as a reflection molecule. The molecules are classified according to the balance between their reaching positions and the set position of the boundaries as shown in Fig 1b. In our previous study, we applied this method in a multi-component system to construct the KBCs for vapor and non-condensable gas molecules [13]. Because the position of the vapor boundary is related to the position where the KBC is applied, the definition of the position of the vapor boundary has already been established from its relationship with the framework of MGD [8, 12, 27]. Hence, we set the vapor boundary at the position of the KBC. On the other hand, we had determined the position of the liquid boundary [12] such that the value of the mass flux of the evaporation molecules coincides with that in the virtual-vacuum condition proposed by Ishiyama et al. [9, 14].

In the classification method that involves setting the two boundaries, the molecules are classified only by their reaching position, and the time that molecules stayed in the vicinity of the vapor–liquid interface is not included in the classification criteria [12]. Molecules that return to the vapor phase without passing through the liquid boundary are classified as the reflection molecules in this method, even if they stayed in the vicinity of the liquid surface for a long time. Thus, there is a possibility that molecules that should be classified as condensation molecules are classified as reflection molecules instead. We consider that, for a single component system, reflection molecules should be the molecules that return to the vapor phase with a short stay on the liquid-phase side and have little interaction with liquid molecules. Unless molecules are properly classified when constructing KBCs, accurate MGD analysis of phase change phenomena cannot be conducted. Thus, in this study, we discuss the validity of the set position of the liquid boundary applied in our previous studies [12, 13, 24] by introducing a new concept, the staying time of molecules, which represents the time they stay in the vicinity of the interface. We obtained molecular properties such as the reaching position and the staying time in MD simulations without setting the liquid boundary, and statistically processed them to understand the characteristics of the molecular motions in the vicinity of the vapor–liquid interface. Based on the distribution of the staying time of molecules that reached in the vicinity of the position where the liquid boundary was set in our previous studies, we investigated whether that position is reasonable for the classification of molecules. Moreover, we also conducted an investigation on the molecular characteristics from the standpoints of molecular velocity distributions and the reflection process of molecules. In particular, while considering the velocity distributions of the reflection molecules, we investigated the dependence of the condensation coefficient *α*_c_ on the molecular velocity at which molecules incident from the vapor phase to the liquid phase.

## 2 Method

### 2.1 System condition

**Fig 2:**
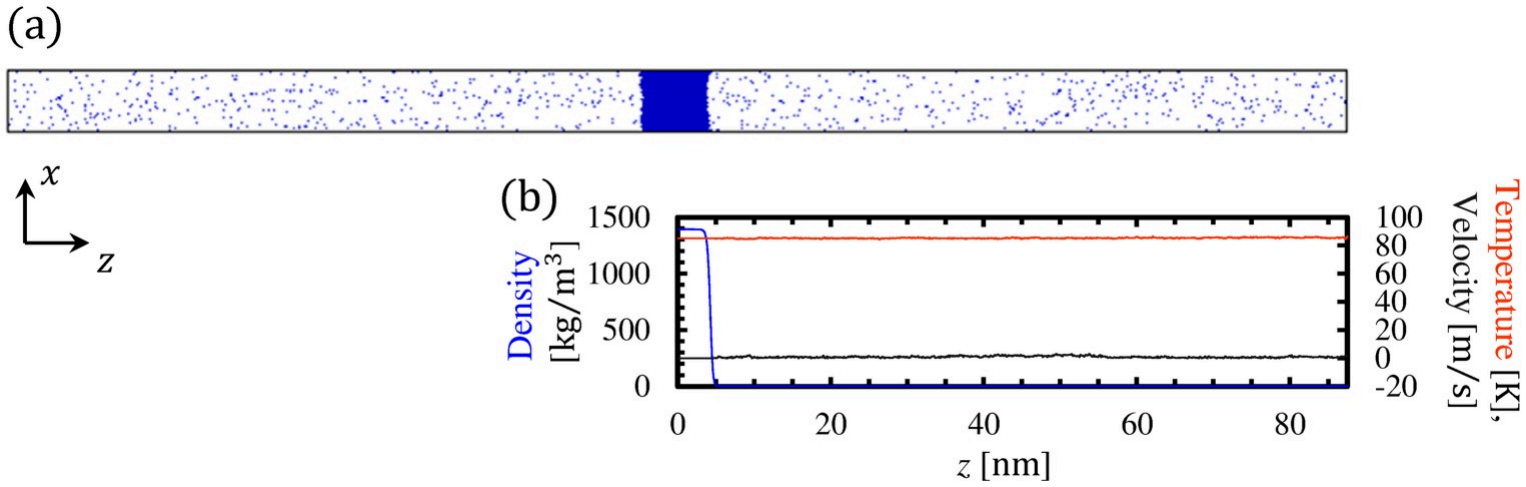
(a) Calculation system in the present MD study; (b) density, temperature, and velocity profiles in system.

We used argon as the liquid and vapor molecules. The calculation domain, which consisted of 12, 000 argon molecules, is shown in Fig 2a. The periodic boundary condition was imposed in all directions of the calculation system. The lengths of the calculation domain *L*_*x*_, *L*_*y*_, and *L*_*z*_ were 8.0, 8.0, and 17.5 nm, respectively.

For the intermolecular potential between argon molecules, we used the following 12-6 Lennard-Jones potential function:

$$\begin{array}{c} \phi_{\text{Ar}}\left(r\right)=4\varepsilon_{\text{Ar}}[{\left(\frac{\sigma_{\text{Ar}}}{r}\right)}^{12}-{\left(\frac{\sigma_{\text{Ar}}}{r}\right)}^{6}], \end{array}$$ (5)

where the molecular diameter *σ*_Ar_ is 0.3405 nm, the potential depth *ε*_Ar_ is 1.635 × 10^−21^ J, and *r* is the distance between two molecules. The cutoff radius was set to 1.5 nm, and the Newton’s law of motion was solved by the leapfrog method with time step Δ*t* = 5 fs.

To establish the equilibrium state for the initial condition of this MD study, we conducted the equilibrium calculation with a temperature control [28] applied to the argon molecules. In the process of this equilibrium calculation, we applied the velocity scaling method at the desired temperature *T* = 85 K. To confirm that the system had reached the equilibrium state, we calculated the density, temperature, and velocity profiles in the system as shown in Fig 2b. These macroscopic values were calculated from the averages for the equilibrium calculation over 200 ns in control volumes with dimensions *L*_*x*_ × *L*_*y*_ × Δ*z*, where Δ*z* = 0.1 nm. Here, the velocity profile is derived from the sum of *x*-, *y*-, and *z*-directional average velocities of molecules. Because the calculation system is symmetric about *z* = 0, the macroscopic values in Fig 2b are obtained by averaging the values on the left (*z* ≤ 0) and right (*z* > 0) parts. We can confirm that the average velocity became 0 m/s and the average temperature became 85 K throughout the calculation system. We have also confirmed that a sum of potential energy of molecules has converged, and since the temperature and velocity profiles are uniform throughout the system, we concluded that it has reached the vapor–liquid equilibrium state at *T* = 85 K. Using this vapor–liquid equilibrium system as the initial condition, we conducted the main simulations for the present study without applying the temperature control.

### 2.2 Definitions of molecular variables

**Fig 3:**
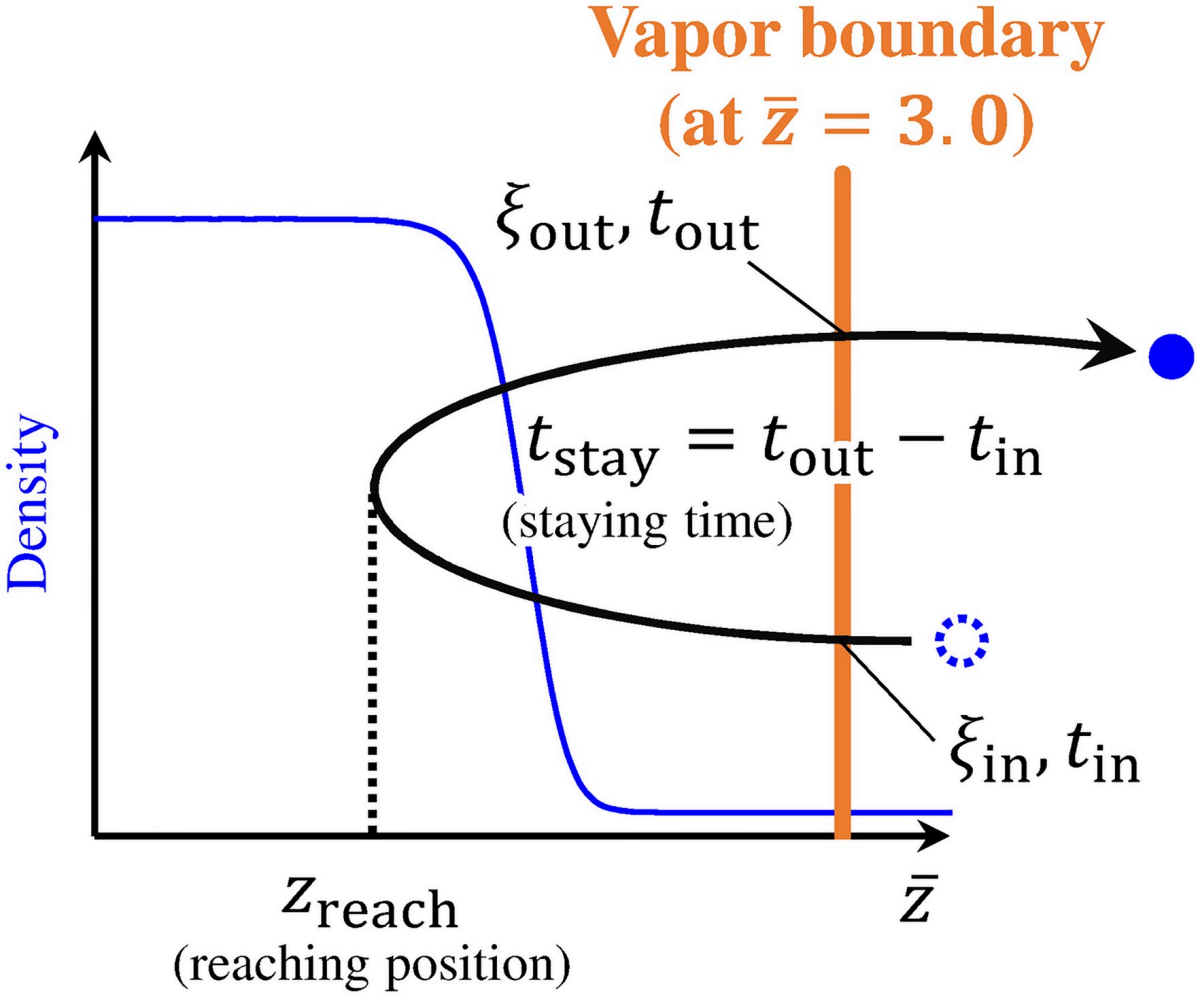
Schematic of method to obtain molecular variables. Reaching position *z*_reach_ was defined as the minimum value of $\bar{z}$ that the molecule had reached; staying time *t*_stay_ was defined as the time that the molecule had stayed in the region on the liquid-phase side of the vapor boundary; *ξ*_in_ and *ξ*_out_ were molecular velocities along the *z*-direction when the molecule passed through the vapor boundary toward the liquid phase and vapor phase, respectively.

In this study, we needed to obtain variables that characterize molecular motions without setting the liquid boundary whose definition is ambiguous. To obtain them, we only set the vapor boundary at the position of the KBC. Here, molecular variables in this study refer to values that can be obtained from molecules based on the position of the vapor boundary. For instance, they include the staying time of molecules which represents a time that a molecule stayed on the liquid-phase side of the vapor boundary (Fig 3). Before giving detailed definitions of molecular variables, we describe the position of the KBC where the vapor boundary is applied.

When specifying the position of the KBC, the following normalized *z* coordinate is commonly utilized:

$$\begin{array}{c} \bar{z}=\frac{z-Z_{m}}{\delta }, \end{array}$$ (6)

where δ is the 10–90 thickness of the density transition layer, and *Z*_m_ is the position of the center of this transition layer. These values were obtained from the hyperbolic tangent function [9, 29] given by

$$\begin{array}{c} \rho \left(z\right)=\frac{\rho_{v}+\rho_{l}}{2}+\frac{\rho_{v}-\rho_{l}}{2}\tanh(\frac{z-Z_{m}}{0.455\delta }), \end{array}$$ (7)

where *ρ*_*v*_ and *ρ*_*l*_ are the densities in the bulk vapor and liquid phases, respectively. In this study, δ became 0.765 nm and *Z*_m_ became 4.24 nm from fitting Eq 7 to the density profile in Fig 2b. We set the vapor boundary at $\bar{z}=3.0$, where the KBCs are widely applied [27].

We obtained variables from the molecules that passed through the vapor boundary toward the liquid phase and passed through the boundary again to return to the vapor phase as shown in Fig 3, because we set only the vapor boundary in this study. From the position of the vapor boundary, we obtained the reaching position *z*_reach_ and the staying time *t*_stay_. The reaching position *z*_reach_ was defined as the minimum value of $\bar{z}$ that a molecule had reached, and the staying time *t*_stay_ was defined as the time that a molecule had stayed on the liquid-phase side of the vapor boundary. In addition to *z*_reach_ and *t*_stay_, we also defined the incident velocity *ξ*_in_ and outgoing velocity *ξ*_out_ as the molecular velocities normal to the vapor boundary (the molecular velocities along the *z*-direction). *ξ*_in_ and *ξ*_out_ were the velocities when a molecule passed through the vapor boundary toward the liquid phase (*ξ*_in_ < 0.0) and toward the vapor phase (*ξ*_out_ > 0.0), respectively. In regard to the condensation and molecular motion, it has been reported that the condensation coefficient of monatomic molecules depends on their translational energy in the direction normal to the liquid surface [30]. Thus, *ξ*_in_ and *ξ*_out_ in this study were defined as the molecular velocities normal to the vapor boundary, and we used them for the investigation of a relationship between *z*-directional velocities of molecules and their condensation or reflection process.

When a molecule returned to the vapor phase, *z*_reach_, *t*_stay_, *ξ*_in_, and *ξ*_out_ were recorded at the same time as samples of the molecule. To obtain a sufficient number of samples of *z*_reach_, *t*_stay_, *ξ*_in_, and *ξ*_out_, we performed the MD simulation in the equilibrium state, in which molecules constantly move toward and away from the vapor–liquid interface. The sampling number of molecules in this study was 100, 000 in all. Because we have confirmed that almost the same results can be obtained when the sampling number of molecules is 10,000, we consider that 100,000 is a sufficient number for the investigation in this study.

Because we had set the liquid boundary at $\bar{z}\approx -1.0$ in our previous studies [12, 13, 24], we will discuss the validity of this position based on the balance between the variables *z*_reach_ and *t*_stay_ of molecules. In particular, we classified the molecules using data clustering as one classification model depending on the two variables *z*_reach_ and *t*_stay_ to verify whether the method that involved setting the two boundaries, which classifies molecules depending only on their reaching position, is a reasonable classification method. We used the *k*-means method of the partitional algorithm for the classification of a dataset [31–33] because it is one of the most popular and the simplest algorithms [34]. The details of the *k*-means method are described in the S1 Appendix. By using data clustering, we can classify a dataset into clusters according to the similarity of the properties of the data points in the dataset. In other words, molecules were classified into clusters based on the similarity of the molecular variables *z*_reach_ and *t*_stay_ using the *k*-means method of data clustering. Owing to the method used to obtain the molecular variables shown in Fig 3, we classified the molecules into two types: molecules with shorter staying times and larger reaching positions, and molecules with longer staying times and smaller reaching positions. From the characteristics of each type of molecules, we considered that the former molecules got reflected and that the latter molecules had condensed once and then evaporated. Thus, we defined the former molecules as reflection molecules and the latter molecules as condensation/evaporation molecules in this study. We classified molecules into reflection molecules and condensation/evaporation molecules by the *k*-means method that includes both *z*_reach_ and *t*_stay_ as variables for clustering. In accordance with the relationship between the variables *z*_reach_ and *t*_stay_ of the molecules and the results of the *k*-means method applied to these variables, we organized the molecular motions in the vicinity of the vapor–liquid interface, and investigated the validity of the position of the liquid boundary at $\bar{z}\approx -1.0$.

## 3 Results and discussion

### 3.1 Reaching position and staying time of molecules

We first describe the features of the dataset representing the relationship between the *z*_reach_ and *t*_stay_ of molecules. Subsequently, we show the results of the *k*-means method that classified molecules into reflection molecules and condensation/evaporation molecules as a model of classification that depends on the two variables *z*_reach_ and *t*_stay_.

**Fig 4:**
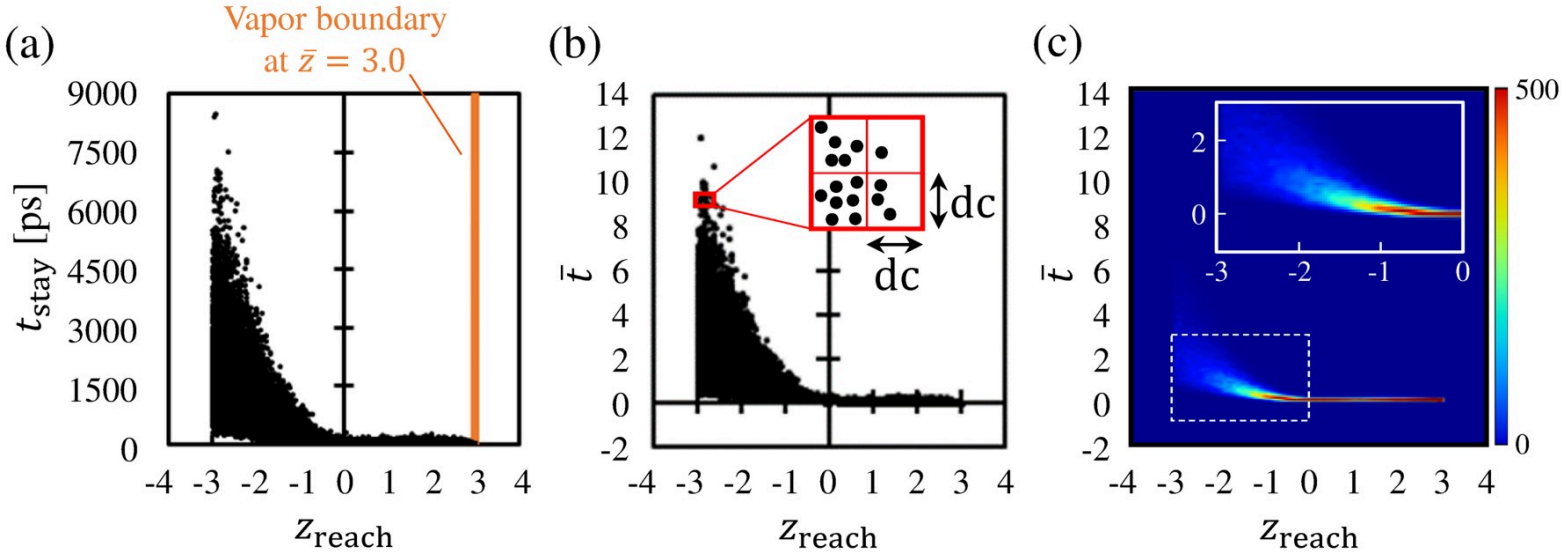
Relationship between *z*_reach_ and *t*_stay_ of molecules in −3.0 < *z*_reach_ < 3.0. (a) Dataset representing relationship between *z*_reach_ and *t*_stay_ of molecules; (b) dataset in *z*_reach_–$\bar{t}$ coordinate plane. Bins with dc × dc are set to calculate the values of the bivariate histogram. (c) Bivariate histogram of data points in *z*_reach_–$\bar{t}$ coordinate plane.

Fig 4a shows the dataset representing the relationship between the *z*_reach_ and *t*_stay_ of the molecules. A data point in this figure denotes the *z*_reach_ and *t*_stay_ of a molecule. Because we aimed to classify molecules in the vicinity of the vapor–liquid interface, we excluded the data points of molecules that reached the deeper bulk liquid phase. To align the distance from the center of the transition layer of the vapor–liquid interface located at $\bar{z}=0.0$, we show the data points of molecules whose *z*_reach_ values were in the range $-3.0<\bar{z}<3.0$ in Fig 4a. The remaining number of data points is 71, 319 out of 100, 000. This figure shows that the molecules that did not pass through the vapor–liquid interface (the molecules with 0.0 < *z*_reach_ < 3.0) returned with a short staying time *t*_stay_. This indicates that *t*_stay_ did not increase when the molecules were reflected in the vapor phase. On the contrary, there is a large variation in the distribution of the *t*_stay_ of molecules whose *z*_reach_ values were within *z*_reach_ < 0.0. This means that *t*_stay_ tends to increase when molecules pass through the vapor–liquid interface and reach the liquid phase. The average *t*_stay_ values of molecules with *z*_reach_ ≥ 0.0 and *z*_reach_ < 0.0 are approximately 25.8 ps and 7.40 × 10^2^ ps, respectively. We consider that this significant increase in *t*_stay_ was due to condensation into the liquid phase. From the variation in *t*_stay_ shown in Fig 4a, we can confirm that the tendency of the *t*_stay_ of molecules changes depending on whether the molecules passed through the vapor–liquid interface.

Although Fig 4a shows the outline of the variation in the data points, the distribution of the number of data points in this figure is unclear. Thus, we constructed the bivariate histogram of the data points in this dataset. We set bins with dc × dc as shown in Fig 4b to count the numbers of data points for the construction of the histogram. To approximately unify the scales of dc in the *t*_stay_ and *z*_reach_ directions, we introduced the non-dimensional $\bar{t}$ coordinate given by

$$\begin{array}{c} \bar{t}=\frac{t_{\text{stay}}}{\mu_{t}}, \end{array}$$ (8)

where *μ*_*t*_ = 6.99 × 10^2^ ps is the standard deviation of *t*_stay_. This *μ*_*t*_ was derived from the *t*_stay_ of molecules whose *z*_reach_ values were within −3.0 < *z*_reach_ < 3.0. We utilized the *z*_reach_–$\bar{t}$ coordinate plane shown in Fig 4b to construct the histogram, and we set dc to 0.1 (to form a balance between the resolution of the histogram and the number of data points). Fig 4c shows the bivariate histogram of data points in the *z*_reach_–$\bar{t}$ coordinate plane. The histogram is represented as the gradation image, and the inset of Fig 4c shows the region surrounded by the dotted rectangle. The color bar denotes number of data points in each bin. As shown in Fig 4c, the bivariate histogram exhibits higher values in 0.0 < *z*_reach_ < 3.0, which is the region between the vapor–liquid interface and the vapor boundary, and it exhibits quite small values in *z*_reach_ < −2.0, which is the region inside the bulk liquid phase. In addition, we can confirm that the values in the bivariate histogram gradually change in the region −2.0 < *z*_reach_ < 0.0. This means that the molecular characteristic in the relationship between *z*_reach_ and *t*_stay_ changes around −2.0 < *z*_reach_ < 0.0, which is the region in the liquid-phase side of the vapor–liquid interface. We consider that this result supports the validity of setting the liquid boundary for the classification of molecules somewhere within this range.

**Fig 5:**
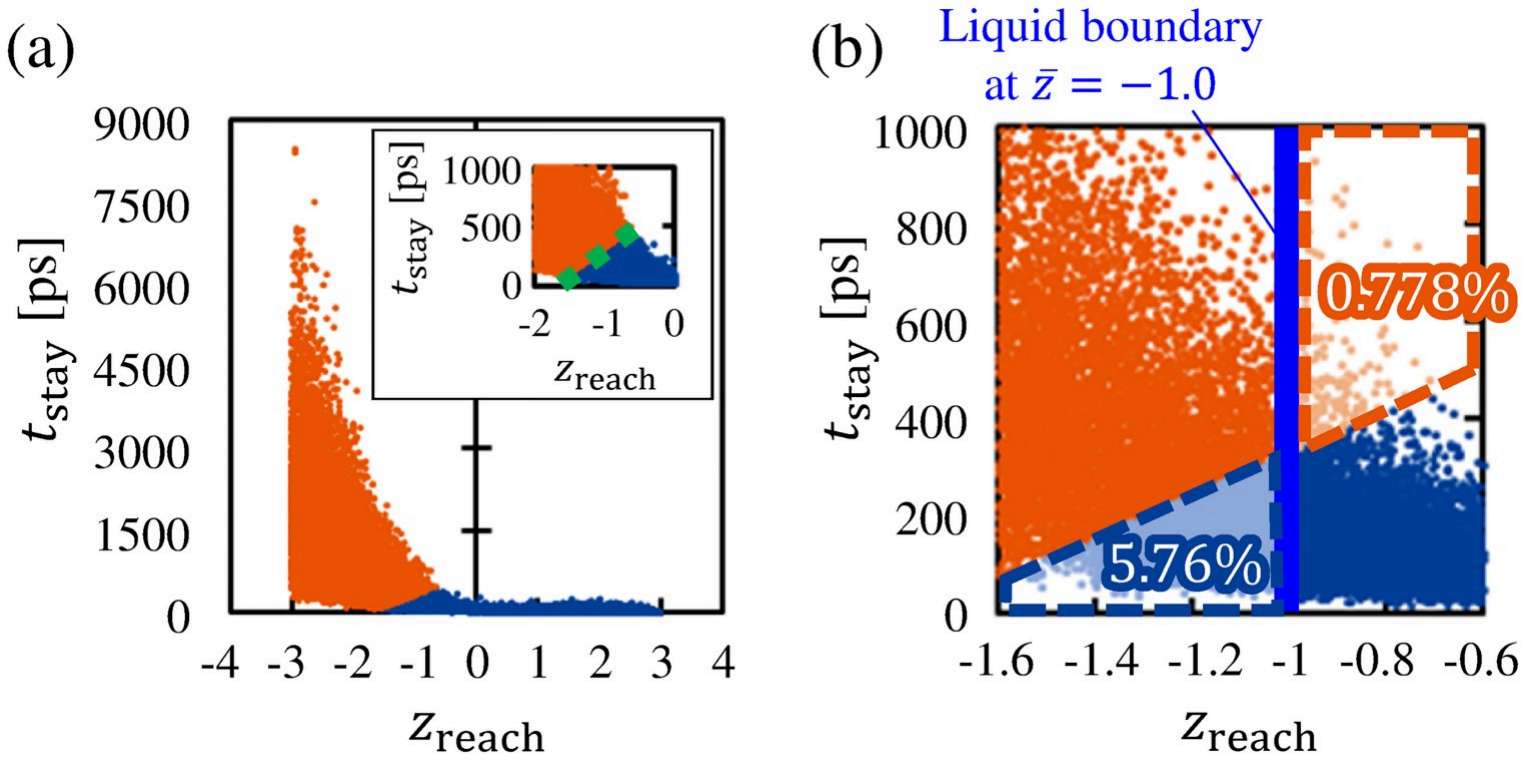
Results of *k*-means method. (a) Dataset classified into two clusters. Blue dots represent the cluster of reflection molecules, and orange dots represent the cluster of condensation/evaporation molecules. Inset shows that the two clusters are divided by a dotted line at approximately *z*_reach_ = −1.0. (b) Percentages of excluded molecules from each cluster in the case where the liquid boundary was set at $\bar{z}=-1.0$.

We next show a classification model depending on the two variables *z*_reach_ and *t*_stay_ yielded by the *k*-means method. The dataset is classified into clusters according to the position of the centroids of data points by the *k*-means method. For details about this method, see the S1 Appendix. The results from the *k*-means method for classifying the molecules into two clusters is shown in Fig 5a. In this figure, the molecules are classified into two clusters: (1) a cluster of molecules with shorter *t*_stay_ and larger *z*_reach_ represented by the blue dots, and (2) a cluster of molecules with longer *t*_stay_ and smaller *z*_reach_ represented by the orange dots. From the characteristics of each cluster, the former was defined as the cluster of the reflection molecules, and the latter was defined as the cluster of condensation/evaporation molecules, as explained in the “Method” section. The numbers of the reflection molecules and condensation/evaporation molecules in this figure are 44, 727 and 26, 592, respectively. The inset of Fig 5a shows the region −2.0 < *z*_reach_ < 0.0, which is the vicinity of the boundary between the two clusters, and the dotted line demarcates the boundary between them. Fig 5a and its inset indicate that the molecules were divided into two clusters at approximately *z*_reach_ = −1.0. To be specific, the dotted line, which represents the boundary between the two clusters, has a slope in approximately the range of −1.5 < *z*_reach_ < −0.5. Most of the range where the bivariate histogram of Fig 4c takes high values is composed of reflection molecules, and their average *t*_stay_ and that of the condensation/evaporation molecules are approximately 52.0 ps and 1.04 × 10^3^ ps, respectively. Because the dataset is classified according to the position of the centroids of the data points in the *k*-means method, the slope of the dotted line in the inset of Fig 5a was caused by the large difference in the *t*_stay_ component of the centroids of the two clusters.

The molecules were classified on the basis of the two variables *z*_reach_ and *t*_stay_ in the *k*-means method, unlike the classification method that involves setting the two boundaries. To compare the classification results obtained from the *k*-means method and the method that involves setting the two boundaries (vapor and liquid boundaries), we calculated the difference in the numbers of molecules classified by the two classification methods. As we conduct the analysis about the liquid boundary set at $\bar{z}\approx -1.0$, we show the case where the liquid boundary is set at $\bar{z}=-1.0$. Fig 5b shows the percentages of molecules excluded by the liquid boundary from the clusters to which they originally belonged. As shown in this figure, approximately 5.76% of the reflection molecules and approximately 0.778% of the condensation/evaporation molecules are excluded from each cluster. Although there are some differences in the resulting classifications, the *k*-means method yielded a similar classification as that produced by the method that involved setting the two boundaries utilized in previous studies. From this result, we consider that it is not necessary to include *t*_stay_ in the classification criteria, if most of the molecules with short *t*_stay_ are classified as the reflection molecules by the liquid boundary set at $\bar{z}=-1.0$.

### 3.2 Staying time and position of liquid boundary

As discussed earlier, the bivariate histogram shown in Fig 4c and the clustering result shown in Fig 5 indicate some validity of the setting of the liquid boundary at $\bar{z}=-1.0$. In this subsection, we investigate the distribution of *t*_stay_ of molecules with *z*_reach_ ≤ −1.0 and *z*_reach_ > −1.0 to confirm whether molecules with short *t*_stay_ are appropriately classified as reflection molecules when the liquid boundary is set at $\bar{z}=-1.0$.

**Fig 6:**
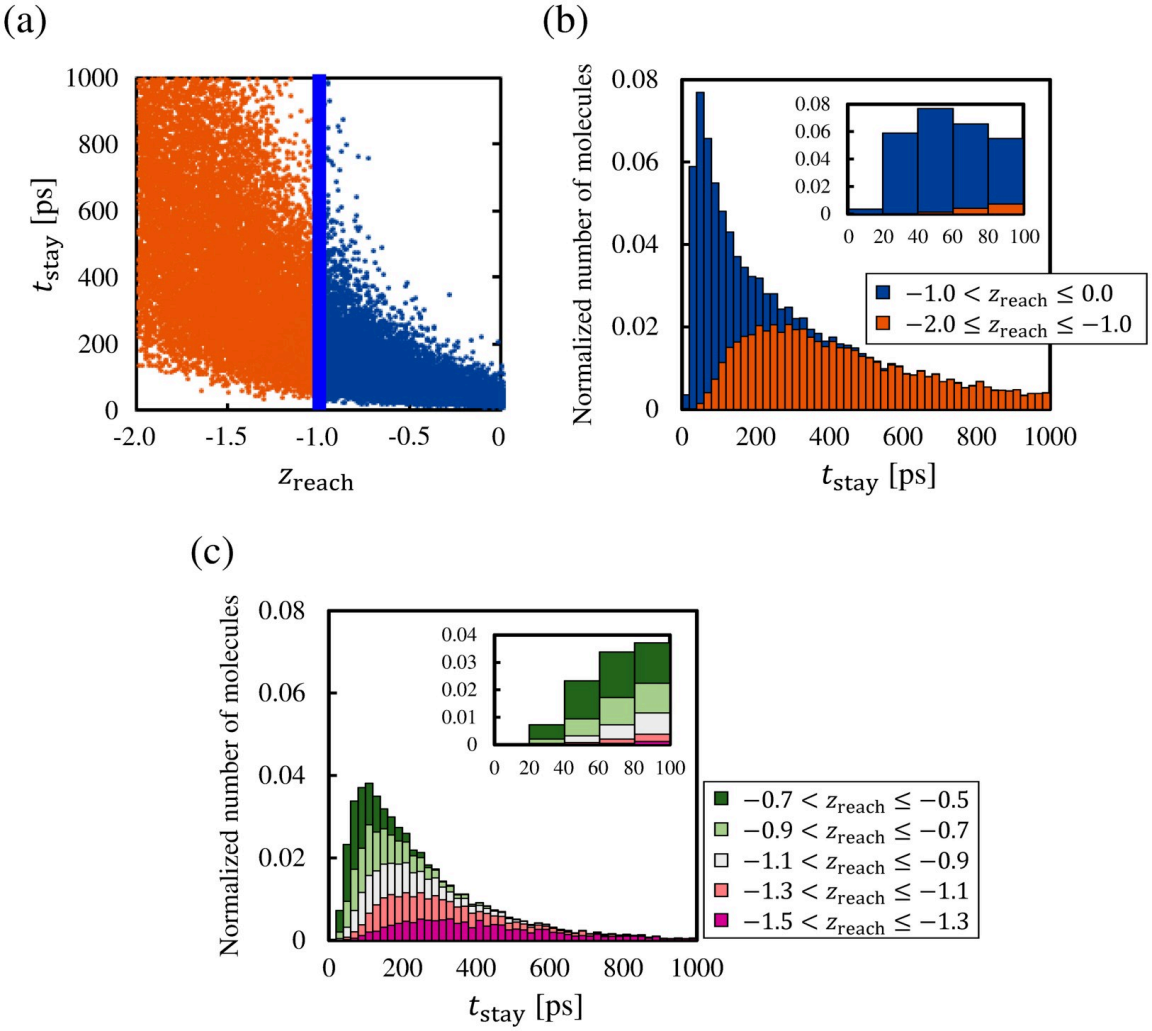
(a) Data points in −2.0 ≤ *z*_reach_ ≤ 0.0 and 0 ps ≤ *t*_stay_ ≤ 1000 ps. (b) Distribution of *t*_stay_ of molecules with −2.0 ≤ *z*_reach_ ≤ 0.0. (c) Distribution of *t*_stay_ of molecules with −1.5 < *z*_reach_ ≤ −0.5. Bar graphs with the time interval 20 ps are composed of the normalized number of molecules in each range of *z*_reach_.

Fig 6a shows the dataset for *z*_reach_ and *t*_stay_ within the ranges −2.0 ≤ *z*_reach_ ≤ 0.0 and 0 ps ≤ *t*_stay_ ≤ 1000 ps. The molecules with −2.0 ≤ *z*_reach_ ≤ −1.0 and −1.0 < *z*_reach_ ≤ 0.0 are represented by the orange dots and blue dots, respectively. The distribution of the *t*_stay_ of these molecules is shown in Fig 6b, and the inset of Fig 6b shows the distribution of *t*_stay_ in *t*_stay_ ≤ 100 ps. The cumulative bar graphs with the time interval Δ*t*_stay_ = 20 ps denote the sum of the normalized numbers of molecules in the two *z*_reach_ ranges. The numbers of molecules are normalized by 25, 366, which is the total number of molecules with −2.0 ≤ *z*_reach_ ≤ 0.0. Fig 6a and 6b show that many of the molecules with −1.0 < *z*_reach_ ≤ 0.0 had *t*_stay_ in the range around *t*_stay_ < 200 ps, whereas the molecules with −2.0 ≤ *z*_reach_ ≤ −1.0 had a widely distributed *t*_stay_ around *t*_stay_ > 100 ps. We can also confirm that there is a peak in the short *t*_stay_ range around *t*_stay_ < 60 ps. Here, it takes approximately 32.6 ps for a molecule to reciprocate the range $-1.0<\bar{z}<3.0$ at the most probable speed $\sqrt{2RT_{L}}$ of the Maxwellian, and the order of this time coincides with *t*_stay_ around the peak in the distribution of Fig 6b. Thus, we consider that the molecules with *t*_stay_ less than 100 ps should be classified as reflection molecules because of their early return to the vapor phase. From Fig 6b and its inset, we can see that the molecules with −1.0 < *z*_reach_ ≤ 0.0 occupy a large part of the range *t*_stay_ < 100 ps. In fact, approximately 95% of molecules with *t*_stay_ < 100 ps have the *z*_reach_ values within −1.0 < *z*_reach_ ≤ 0.0. This indicate that most of the molecules with a fairly short *t*_stay_ are classified as reflection molecules when we classify the molecules based on whether their *z*_reach_ is less than −1.0, that is, by setting the liquid boundary at $\bar{z}=-1.0$. Thus, we conclude that $\bar{z}=-1.0$ applied in our previous studies is the reasonable position of the liquid boundary for the classification of molecules.

In addition, we investigate whether there is a more suitable position of the liquid boundary at around $\bar{z}=-1.0$. Fig 6c shows the distribution of *t*_stay_ of molecules with −1.5 < *z*_reach_ ≤ −0.5. We divided this *z*_reach_ range into five, and the cumulative bar graphs with the time interval Δ*t*_stay_ = 20 ps denote the sum of the normalized numbers of molecules in each *z*_reach_ range. The numbers of molecules are normalized by 25, 366, which is the total number of molecules with −2.0 ≤ *z*_reach_ ≤ 0.0. The inset of Fig 6c shows the distribution of *t*_stay_ in the range *t*_stay_ ≤ 100 ps. As shown in Fig 6c, there is a non-negligible number of molecules with −0.9 < *z*_reach_ ≤ −0.5 in the short *t*_stay_ range (*t*_stay_ < 100 ps). Approximately 91% of molecules with *t*_stay_ < 100 ps have the *z*_reach_ values within −0.9 < *z*_reach_ ≤ 0.0, and it indicates that almost the same classification as when the liquid boundary is set at $\bar{z}=-1.0$ can be obtained by setting the liquid boundary at $\bar{z}=-0.9$. From the above results, we found that most of the molecules with a fairly short staying time (*t*_stay_ < 100 ps) are classified as reflection molecules when the liquid boundary is set at $\bar{z}=-0.9$ or −1.0. Therefore, we conclude that the suitable position of the liquid boundary for the classification of molecules is $-1.0\leq \bar{z}\leq -0.9$.

### 3.3 Incident and outgoing velocities of reflection molecules

**Fig 7:**
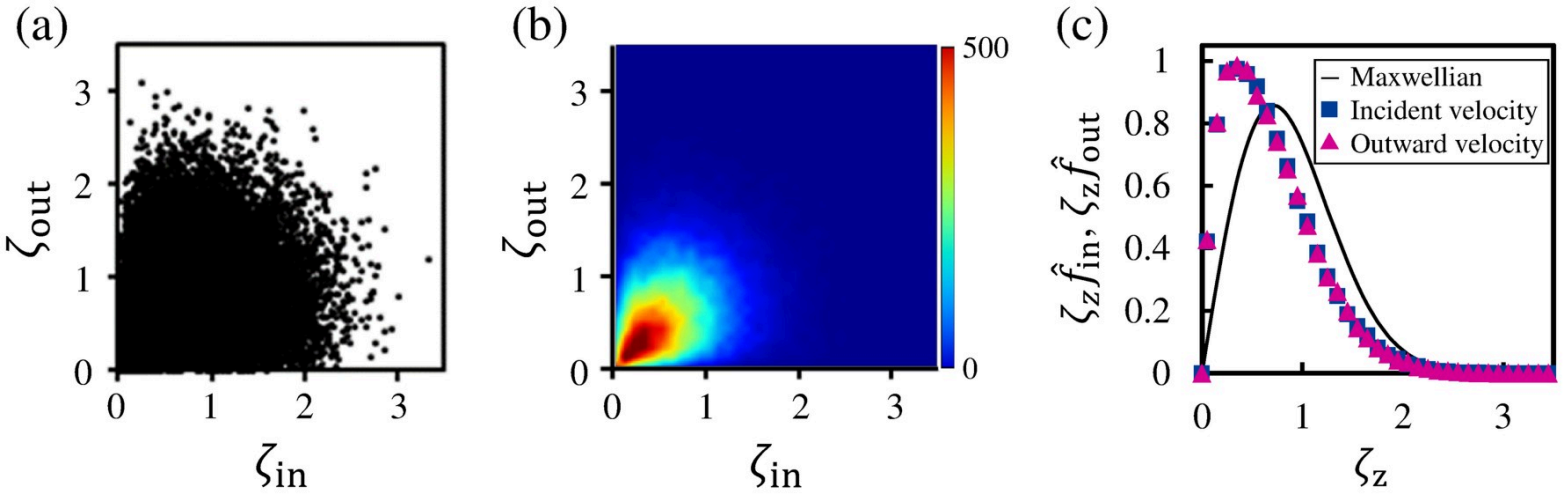
Relationship between normalized molecular velocities *ζ*_in_ and *ζ*_out_ of reflection molecules. (a) Data points representing the relationship between *ζ*_in_ and *ζ*_out_ of reflection molecules. Sampling number of reflection molecules is 42, 359. (b) Bivariate histogram in the *ζ*_in_–*ζ*_out_ coordinate plane. (c) Molecular velocity distribution functions of reflection molecules for the normalized incident velocity *ζ*_in_ and normalized outgoing velocity *ζ*_out_.

In this subsection, we investigate the characteristics of reflection molecules in terms of their incident and outgoing velocities. From this subsection, we define the molecules with *z*_reach_ ≤ −1.0 as condensation/evaporation molecules and those with −1.0 < *z*_reach_ < 3.0 as reflection molecules. The numbers of reflection molecules and condensation/evaporation molecules according to this definition are 42, 359 and 57, 641, respectively. The dataset representing the relationship between the incident velocity and the outgoing velocity of reflection molecules is shown in Fig 7a. *ζ*_in_ in the abscissa and *ζ*_out_ in the ordinate are the normalized molecular velocities $\zeta_{\text{in}}=\left|\xi_{\text{in}}\right|/\sqrt{2RT_{L}}$ and $\zeta_{\text{out}}=\xi_{\text{out}}/\sqrt{2RT_{L}}$, respectively. $\sqrt{2RT_{L}}$ denotes the most probable speed of the Maxwellian at the liquid temperature *T*_L_ = 85 K. The incident velocity *ξ*_in_, which was in the negative *z*-direction as shown in Fig 3, was set to be positive for comparison with the outgoing velocity *ζ*_out_ in the same coordinate system. To investigate the distribution of the data points, we constructed the bivariate histogram of this dataset as shown in Fig 7b. The bivariate histogram in the *ζ*_in_–*ζ*_out_ coordinate plane is constructed and represented using the same method that involves setting the bins with dc × dc = 0.1 × 0.1 as in Fig 4c. We can confirm that the bivariate histogram shows the high values in the ranges 0 < *ζ*_in_ < 1.0 and 0 < *ζ*_out_ < 1.0 in Fig 7b. Approximately 64% of reflection molecules had the incident and outgoing velocities in these velocity ranges, and it means that they had lower *ζ*_in_ and *ζ*_out_ than the most probable speed of the Maxwellian *ζ*_*z*_ = 1.0.

Fig 7c shows the velocity distribution functions for the incident and outgoing velocities of the reflection molecules. The velocity distributions in this figure are shown as functions of $\zeta_{z}{\hat{f}}_{\text{in}}$ and $\zeta_{z}{\hat{f}}_{\text{out}}$, where ${\hat{f}}_{\text{in}}$ and ${\hat{f}}_{\text{out}}$ are the normalized velocity distribution functions of the incident velocity and outgoing velocity, respectively. The solid line represents the normalized Maxwellian at 85 K given by $\zeta_{z}{\hat{f}}^{*}=2\zeta_{z}\exp\left(-\zeta_{z}^{2}\right)$, where ${\hat{f}}^{*}$ is the normalized velocity distribution function of molecules in the vapor–liquid equilibrium state. As shown in Fig 7c, the incident and outgoing velocities show almost the same velocity distribution functions. Fig 7c also indicates that the velocity distribution functions of $\zeta_{z}{\hat{f}}_{\text{in}}$ and $\zeta_{z}{\hat{f}}_{\text{out}}$ exhibit large deviations from the Maxwellian. The peaks of these distributions are formed at approximately *ζ*_*z*_ = 0.4, which is a smaller velocity than the mean velocity of the Maxwellian, *ζ*_m_ ≈ 0.707. This means that in the velocity distributions of reflection molecules, the ratio of molecules with low velocities is higher than that in the Maxwellian. Therefore, it can be said that molecules with a low incident velocity are easily reflected. In addition, from the deviation of the velocity distribution function for the outgoing velocity from the Maxwellian, we can confirm that many of the reflection molecules pass through the vapor boundary toward the vapor phase without being accelerated to their *z*-directional velocity after reflection.

Basically, the velocity distribution function of all incident molecules follows the Maxwellian in the vapor–liquid equilibrium state. The reflection of molecules with a lower incident velocity indicates the possibility that molecules with a higher incident velocity will reach the liquid phase. Therefore, we next investigate the relationship between the reaching position *z*_reach_ and the incident velocity *ξ*_in_ of molecules.

### 3.4 Reaching position and incident velocity

**Fig 8:**
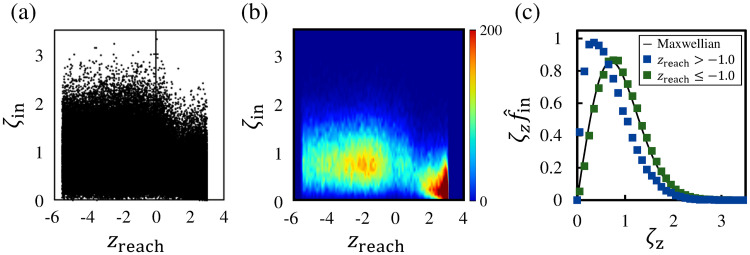
Relationship between *z*_reach_ and normalized incident velocity *ζ*_in_ of molecules. (a) Data points representing the relationship between *z*_reach_ and *ζ*_in_ of all sampling molecules. (b) Bivariate histogram in the *z*_reach_–*ζ*_in_ coordinate plane. (c) Molecular velocity distribution functions of reflection molecules whose *z*_reach_ values lie in *z*_reach_ > −1.0 and condensation/evaporation molecules whose *z*_reach_ values lie in *z*_reach_ ≤ −1.0.

The dataset representing the relationship between *z*_reach_ and the normalized incident velocity *ζ*_in_ of molecules is shown in Fig 8a, and the bivariate histogram of this dataset is shown in Fig 8b. The bivariate histogram is constructed using the same method that involves setting the bins with dc × dc = 0.1 × 0.1 as in Fig 4c. Although we excluded the data points of molecules with *z*_reach_ < −3.0 in the previous subsections, the data points of all sample molecules, including the molecules with *z*_reach_ ≤ −3.0 that reached the deep bulk liquid phase, are shown in these figures for elucidating the dependence of the *z*_reach_ of molecules on their incident velocities. Thus, the sample number of molecules in Fig 8 is 100, 000. As shown in Fig 8b, many of the molecules with a low incident velocity were reflected in the vicinity of the vapor boundary at $\bar{z}=3.0$. The average incident velocity of molecules whose *z*_reach_ values were within 2.0 < *z*_reach_ < 3.0 is *ζ*_in_ = 0.459, and it indicates that molecules with low incident velocities promptly return to the vapor phase. Furthermore, approximately 65% of the molecules with *ζ*_in_ > 0.5 had *z*_reach_ values within *z*_reach_ ≤ −1.0, and it means that many of the molecules with not so low *ζ*_in_ are easy to reach the liquid phase and condense into it.

We next compare the incident velocity of the reflection molecules and that of the condensation/evaporation molecules using the velocity distribution functions. Fig 8c shows the velocity distribution functions for the incident velocity of the reflection molecules with *z*_reach_ > −1.0 and the condensation/evaporation molecules with *z*_reach_ ≤ −1.0. As in Fig 7c, the velocity distribution functions are shown as a function of $\zeta_{z}{\hat{f}}_{\text{in}}$. The solid line represents the Maxwellian at 85 K given by $\zeta_{z}{\hat{f}}^{*}$, and the velocity distribution function of the reflection molecules in Fig 8c is identical to that in Fig 7c. As shown in Fig 8c, the velocity distribution function of the condensation/evaporation molecules is almost consistent with the Maxwellian, unlike that of the reflection molecules. The incident velocity in the region where the bivariate histogram shows the higher values in $\bar{z}<-1.0$ in Fig 8b corresponds to the mean velocity of the Maxwellian. This means that the molecules basically reach $\bar{z}<-1.0$ and condense into the liquid phase when their incident velocities are nearly equal to the mean velocity of the Maxwellian. Fig 8 demonstrates that the incident velocity greatly influences the reaching position *z*_reach_, i.e., the condensability of molecules, and we conclude that the condensation of molecules depends on their incident velocity. However, the dependence of the condensation coefficient *α*_c_, which is defined as the ratio of the molecular mass fluxes, on the incident velocity of molecules has not been investigated. Thus, in the next subsection, we discuss *α*_c_ and its relationship with the molecular incident velocity.

### 3.5 Condensation coefficient and incident velocity

#### 3.5.1 Dependence of condensation coefficient on incident velocity

We first show the relationship between *α*_c_ and molecular mass fluxes in detail. From Eqs 3 and 4, *α*_c_ is given by

$$\begin{array}{c} \alpha_{c}=1-\frac{J_{\text{ref}}}{J_{\text{coll}}}. \end{array}$$ (9)

We assume that the mass flux of colliding molecules *J*_coll_ in the vapor–liquid equilibrium state is given by

$$\begin{array}{c} J_{\text{coll}}=-\int_{-\infty }^{0}\int_{-\infty }^{\infty }\int_{-\infty }^{\infty }\xi_{z}f^{*}\;d\xi_{x}d\xi_{y}d\xi_{z}=\rho^{*}\sqrt{\frac{RT_{L}}{2\pi }}, \end{array}$$ (10)

where f* is the Maxwellian at a liquid temperature *T*_L_. Combining Eqs 9 and 10, we can derive *α*_c_ as

$$\begin{array}{c} \alpha_{c}=1-\frac{J_{\text{ref}}}{\rho^{*}\sqrt{RT_{L}/2\pi }}. \end{array}$$ (11)

**Fig 9:**
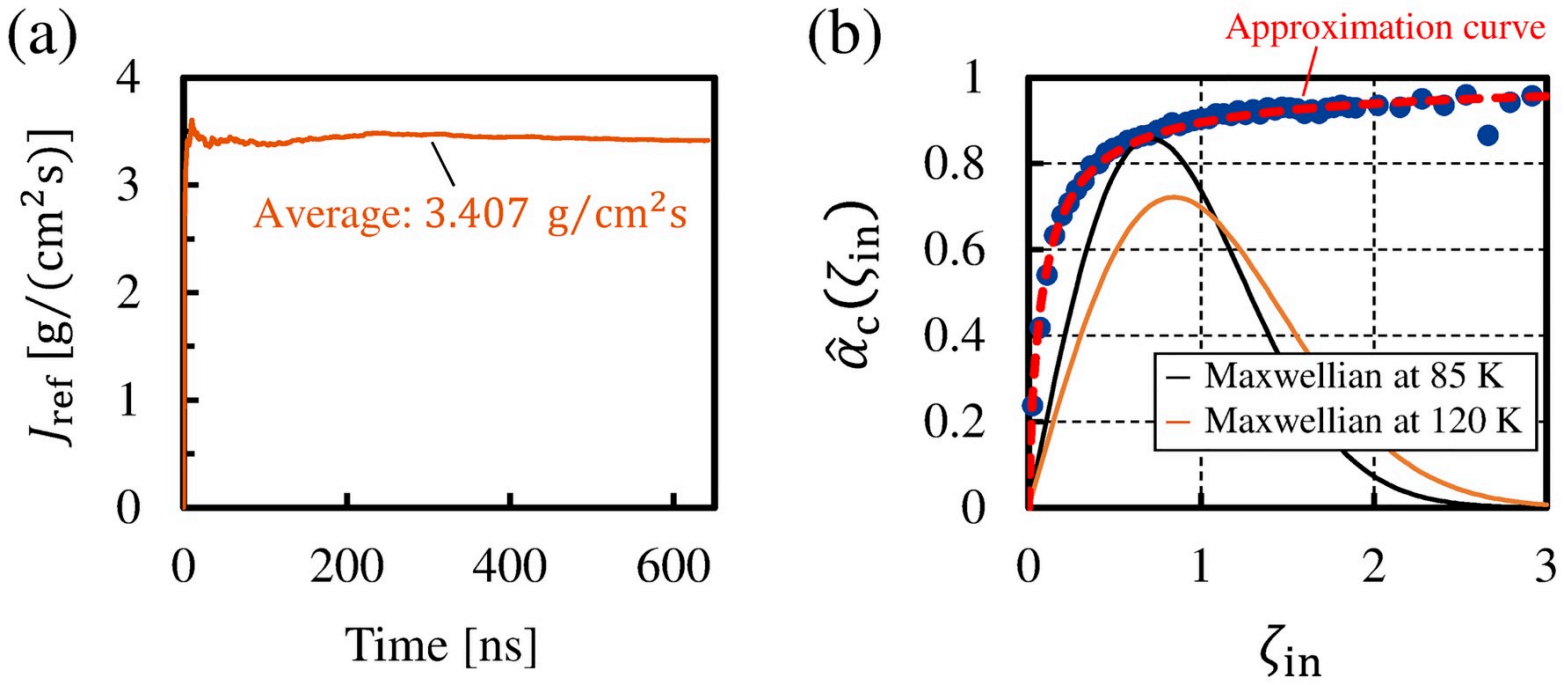
(a) Time evolution of *J*_ref_ in the present MD study; (b) condensation coefficient for each range of incident velocity *ζ*_in_.

From this equation, we can calculate *α*_c_ from the mass flux *J*_ref_ and the saturated vapor density ρ*. Fig 9a shows the time evolution of *J*_ref_ throughout the MD simulation in this study. The value of the mass flux reaches a certain constant value, although it fluctuates in the first stage of the simulation. From the time average of the value of the mass flux, we obtained *J*_ref_ = 3.407 g/cm^2^ s. Further, the density field of the argon molecules in the calculation system shown in Fig 2 provided the saturated vapor density as ρ* = 4.761 kg/m^3^. This ρ* is calculated from the average density in the region of the bulk vapor phase. From these values, we obtained *α*_c_ in the present MD simulation as *α*_c_ = 0.865. This value of *α*_c_ agrees well with that calculated in the previous studies [10, 13]. Hence, we concluded that *α*_c_ in this study was correctly calculated from the *J*_ref_ obtained by this MD simulation and the *J*_coll_ obtained by Eq 10.

We next investigate *α*_c_ in detail using Eqs 10 and 11. As we discussed in the previous subsection, the molecules reflected in the vicinity of the vapor boundary had low incident velocities. Moreover, Fig 8b shows that many of the molecules whose incident velocities were nearly equal to the mean velocity of the Maxwellian reached and condensed into the liquid phase. These results represent that the condensability of molecules depends on their incident velocity at the liquid phase. In fact, it has been reported that the condensation coefficient shows the low value for the small molecular translational energy in the normal component to the liquid surface in the previous studies [30, 35, 36]. However, in these previous studies, the definitions of the condensation of molecules and the condensation coefficient were different from the definitions used in the present study. Hence, to confirm the dependency of *α*_c_ on the incident velocity of molecules based on the definition of the present study, we calculated *α*_c_ for each incident velocity range.

From Eq 10, the mass flux of colliding molecules with an incident velocity within the range *A* < *ξ*_*z*_ < *B* is given by

$$\begin{array}{c} J_{\text{coll}}^{A-B}=-\int_{A}^{B}\int_{-\infty }^{\infty }\int_{-\infty }^{\infty }\xi_{z}f^{*}\;d\xi_{x}d\xi_{y}d\xi_{z},\;\text{for}\;B<0. \end{array}$$ (12)

The velocity interval Δ*ξ*_*z*_ for the calculation was set to Δ*ξ*_*z*_ = *B* − *A* = 8 m/s. We also calculated $J_{\text{ref}}^{A-B}$ from the incident velocities *ξ*_in_ of reflection molecules within the range *A* < *ξ*_*z*_ < *B*, which were obtained in the present MD simulation. By assigning $J_{\text{coll}}^{A-B}$ and $J_{\text{ref}}^{A-B}$ to Eq 9, the condensation coefficient ${\hat{\alpha }}_{c}$ of molecules with incident velocities in *A* < *ξ*_*z*_ < *B* is given by

$$\begin{array}{c} {\hat{\alpha }}_{c}=1-\frac{J_{\text{ref}}^{A-B}}{J_{\text{coll}}^{A-B}}. \end{array}$$ (13)

Fig 9b shows ${\hat{\alpha }}_{c}$ for each range of the normalized incident velocity *ζ*_in_. The condensation coefficient is found to be a function of the molecular incident velocity ${\hat{\alpha }}_{c}\left(\zeta_{\text{in}}\right)$ from this figure. We can see that the value of ${\hat{\alpha }}_{c}\left(\zeta_{\text{in}}\right)$ increases as *ζ*_in_ increases, and ${\hat{\alpha }}_{c}\left(\zeta_{\text{in}}\right)$ sensitively responds to *ζ*_in_ when *ζ*_in_ is less than the most probable speed in the Maxwellian (*ζ*_in_ < 1.0). This figure also shows that ${\hat{\alpha }}_{c}\left(\zeta_{\text{in}}\right)$ becomes almost constant when *ζ*_in_ < 1.0 although ${\hat{\alpha }}_{c}\left(\zeta_{\text{in}}\right)$ with *ζ*_in_ > 2.0 fluctuate due to the small number of sample molecules. The molecules with low *ζ*_in_ show a small value of ${\hat{\alpha }}_{c}\left(\zeta_{\text{in}}\right)$, and it means that they are less likely to condense into the liquid phase than molecules with high *ζ*_in_.

The dashed line in Fig 9b, which represents the approximation curve for ${\hat{\alpha }}_{c}$, is given by

$$\begin{array}{c} {\hat{\alpha }}_{c}\left(\zeta_{\text{in}}\right)=-\frac{C_{1}}{C_{1}+\zeta_{\text{in}}^{C_{2}}}+1, \end{array}$$ (14)

where *C*_1_ = 10.36 and *C*_2_ = 0.8566 under the conditions of the present MD simulation. Because this approximation curve is in good agreement with ${\hat{\alpha }}_{c}$ obtained from Eq 13, the condensation coefficient as the function of *ζ*_in_ can be given by Eq 14 in this study.

Here, we show the relationship between *α*_c_ defined in Eq 11 and ${\hat{\alpha }}_{c}\left(\zeta_{\text{in}}\right)$ defined in Eq 13. As shown in Fig 9b, ${\hat{\alpha }}_{c}\left(\zeta_{\text{in}}\right)$ is the condensation coefficient of the function of the incident velocity. On the other hand, *α*_c_ defined in Eq 11 is the condensation coefficient of the entire incident velocity range −∞ < *ζ*_in_ < 0. Thus, by using ${\hat{\alpha }}_{c}\left(\zeta_{\text{in}}\right)$, *α*_c_ is derived as

$$\begin{array}{c} \alpha_{c}=-\int_{-\infty }^{0}\zeta_{z}{\hat{f}}_{\text{in}}{\hat{\alpha }}_{c}\left(\zeta_{z}\right)\;d\zeta_{z}. \end{array}$$ (15)

We have confirmed that *α*_c_ was calculated as *α*_c_ = 0.864 from this equation, and this value agrees well with the value of *α*_c_ = 0.865 calculated from Eq 11. Thus, we conclude that *α*_c_ was appropriately yielded by Eq 15 with ${\hat{\alpha }}_{c}\left(\zeta_{\text{in}}\right)$ in Fig 9b.

#### 3.5.2 Condensation coefficient in equilibrium state and non-equilibrium state

As defined in Eq 13, ${\hat{\alpha }}_{c}\left(\zeta_{\text{in}}\right)$ was derived from $J_{\text{coll}}^{A-B}$ and $J_{\text{ref}}^{A-B}$, which represent the mass fluxes of the colliding molecules and reflection molecules whose incident velocities were in certain ranges. Because the ratios between these fluxes are uniquely determined by the liquid temperature, the same profile of ${\hat{\alpha }}_{c}\left(\zeta_{\text{in}}\right)$ as in Fig 9b will be obtained regardless of whether the calculation system is the non-equilibrium or equilibrium state when the liquid temperature is fixed as *T*_L_ = 85 K. The molecular velocity distribution function for the incident velocity ${\hat{f}}_{\text{in}}$ changes depending on the system state whereas ${\hat{\alpha }}_{c}\left(\zeta_{\text{in}}\right)$ maintains the same profile in any state with *T*_L_ = 85 K. This means that we can calculate *α*_c_ in the non-equilibrium state with *T*_L_ = 85 K from ${\hat{\alpha }}_{c}\left(\zeta_{\text{in}}\right)$ in Fig 9b and the molecular velocity distribution functions that depend on the system state. Discussions on the relationship between molecular velocity distribution functions and the condensability based on the different definition from that in the present sutudy have been conducted in the previous study [36]. Thus, we next calculate *α*_c_ in the non-equilibrium state with the fixed liquid temperature *T*_L_ = 85 K by assuming a molecular velocity distribution function for the incident velocity ${\hat{f}}_{\text{in}}$, which represents a net condensation of molecules.

In regard to the condensation coefficient *α*_c_ in the non-equilibrium state, Kon et al. reported that the value of *α*_c_ slightly changed when the net condensation occurred in the vapor temperature range of approximately 85–120 K [8]. To confirm whether the same tendency as that in their study can be obtained from ${\hat{\alpha }}_{c}\left(\zeta_{\text{in}}\right)$ in Fig 9b, we calculate *α*_c_ in the vapor temperatures at 85 K and 120 K with the fixed liquid temperature *T*_L_ = 85 K. The two Maxwellians at 85 K and 120 K are shown as the normalized functions of $\zeta_{z}{\hat{f}}^{*}\left(T=85\;K\right)$ and $\zeta_{z}{\hat{f}}^{*}\left(T=120\;K\right)$ in Fig 9b, and the incident velocities *ξ*_in_ in both functions were normalized by the most probable speed of the Maxwellian at *T*_L_ = 85 K. The Maxwellian at *T*_L_ = 120 K assumes non-equilibrium condensation where the incident molecules enter the liquid phase with the velocity distribution function $\zeta_{z}{\hat{f}}^{*}\left(T=120\;K\right)$. As the liquid temperature was set at *T*_L_ = 85 K in this study, the calculation of *α*_c_ from Eq 15 with the molecular velocity distribution function $\zeta_{z}{\hat{f}}^{*}\left(T=85\;K\right)$ yields *α*_c_ = 0.864 as shown before. This value means the condensation coefficient in the equilibrium state. In the case of the non-equilibrium state where the molecular velocity distribution function in Eq 15 was $\zeta_{z}{\hat{f}}^{*}\left(T=120\;K\right)$, *α*_c_ was calculated as 0.878. The value of *α*_c_ obtained from Eq 15 varied slightly as expected when the system was non-equilibrium, and a slight increase in the value of *α*_c_ in the non-equilibrium system was reported in the previous study [8]. However, there is no significant difference between the calculated values of *α*_c_ in each system state. Therefore, although the condensation coefficient depends on the incident velocity of molecules as shown in Fig 9b, we conclude that the value of the condensation coefficient is almost constant regardless of whether the system is equilibrium or non-equilibrium.

We investigated the condensation coefficient *α*_c_ in terms of the incident velocity of molecules with the mass flux of colliding molecules *J*_coll_ in this study. By defining the mass flux of outgoing molecules $J_{\text{out}}^{*}$ within the outgoing velocity range of *A* < *ξ*_*z*_ < *B* in the same way as the $J_{\text{coll}}^{A-B}$ in Eq 12, the discussion in this subsection can be applied to investigate the dependence of the evaporation coefficient *α*_e_ on molecular velocities.

## 4 Conclusion

In this study, we conducted MD simulations to discuss the position of the liquid boundary for the classification of molecules in the vicinity of the vapor–liquid interface to construct the KBCs. Because we had set the liquid boundary at $\bar{z}\approx -1.0$ in our previous studies, we investigated the validity of this position based on the molecular variables *t*_stay_ and *z*_reach_, which characterize their motions in the vapor–liquid interface. To obtain the criterion for classifying molecules depending on these two variables, we used the *k*-means method of data clustering in which molecules were classified into clusters according to the similarity of the two variables. The results of the *k*-means method and the bivariate histogram that represents the relationship between the *t*_stay_ and *z*_reach_ of molecules show that we do not necessarily have to include *t*_stay_ in the classification criteria. Moreover, by considering the distribution of the *t*_stay_ of molecules whose *z*_reach_ values were in the vicinity of the vapor–liquid interface, we confirmed that most of the molecules with short *t*_stay_ values were classified as the reflection molecules when the liquid boundary was set at $\bar{z}\approx -1.0$. Therefore, we concluded that the liquid boundary at $\bar{z}\approx -1.0$ applied in our previous studies yields a reasonable classification of molecules for the construction of KBCs.

Furthermore, we also investigated the relationship between the condensability of molecules and their incident velocities at the liquid phase. We found that molecules with a low incident velocity were reflected in the vicinity of the vapor boundary, and molecule whose incident velocity was near the mean velocity of the Maxwellian basically reached the liquid phase and condensed into it. Because it indicates the dependence of the condensation of molecules on their incident velocity, we calculated the condensation coefficient as a function of the incident velocity: ${\hat{\alpha }}_{c}\left(\zeta_{\text{in}}\right)$. As a result, the value of ${\hat{\alpha }}_{c}\left(\zeta_{\text{in}}\right)$ became small in the low-incident-velocity range, and it showed a constant value when the incident velocity exceeded the most probable speed of the Maxwellian. We also calculated *α*_c_ in the non-equilibrium system where the net condensation occurred, and we confirmed that *α*_c_ had almost the same value as that in the equilibrium system.

## Supporting information

S1 Appendix The *k*-means method. (PDF)
