# Supplementary material for: Molecular dynamics study on characteristics of reflection and condensation molecules at vapor–liquid equilibrium state
Source: PLoS One. 2021 Mar 16;16(3):e0248660. doi: 10.1371/journal.pone.0248660 (PMC7963090; doi:10.1371/journal.pone.0248660)
Supplement: S1 Appendix — (PDF) [file pone.0248660.s001.pdf]

S1 Appendix

The k-means method

The k-means method is one of the most popular partitional clustering method. We can classify a dataset into several clusters by using data clustering to gain further insights into the dataset. In this study, we used the reaching position z_{reach} and staying time t_{stay} of molecules as the variables for clustering. In general, variables are standardized in preparation for the clustering processes to unify their scales or units. An i-th data x_i is standardized by

$$x_i^* = \frac{x_i - m_x}{\mu},$$

where μ is the standard deviation, and m_x is the mean x of all the data points. We standardized the z_{reach} and t_{stay} of the molecules using the above equation. Although we performed the k-means method on standardized variables, we plotted the clustering results in the original coordinates system in the main manuscript because the standardized values were less informative.

We now introduce the details and procedures of the clustering methods. Fig. A shows the concept of the k-means method of the partitional algorithm for the classification of a dataset. First, we have to select the number of clusters k, and k centroids of clusters are placed at random positions as shown in Fig. A(a). In this step, we need to discuss the appropriate number of clusters for classification of data points if the number of clusters had not been decided in advance. In the present study, molecules are to be classified into reflection molecules and condensation/evaporation molecules. Thus, we set the number of clusters k as $k = 2$. In the next step, each data point is assigned to the closest centroid as a cluster, and the position of the centroid of each cluster is recalculated after all data points are assigned as shown in Figs. A(b) and (c). The calculations of the distance between data points and the centroids were based on the Euclidean distance in this study. By iteratively assigning the data points and recalculating the position of the centroids as shown in Figs. A(d)–(g), the classification of data points is obtained. In the k-means method, all data points are assigned to each cluster so that the following sum of the squares between data points and centroids of clusters is minimized:

$$d = \sum_{k=1} \sum_{i \in c_k} \|\boldsymbol{x}_i^* - \boldsymbol{m}_k\|^2,$$

where $\boldsymbol{x}_i^*$ is a standardized variable of a data point i belonging to a cluster c_k, and $\boldsymbol{m}_k$ is the centroid of c_k. As mentioned before, the calculation of this equation was based on the Euclidean distance.

A known problem with the k-means method is that a clustering result can depend on the initial positions of the centroids placed at random points as shown in Fig A(a). This dependence on the initial position appears when the distribution of data points in a dataset has symmetry. In this study, there was no symmetry in the distribution of the data points in the dataset representing the relationship between the z_{reach} and t_{stay} of the molecules (shown in the main manuscript). Hence, it was not necessary to consider this issue with respect to the results of the k-means method in the present study. In fact, we have confirmed that the initial positions of the centroids had no influence on the clustering results in more than 10 attempts of the k-means method.

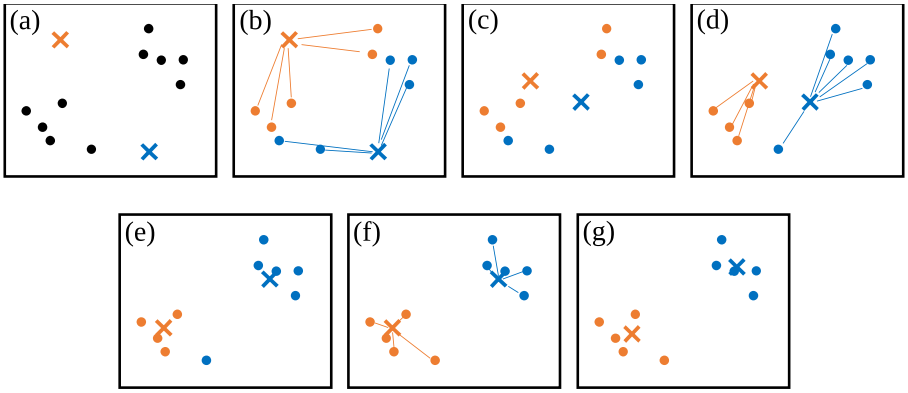

Fig A. Schematics of the clustering process of the *k*-means method. (a) Two centroids indicated by crosses at random points and input data points; (b) assigning data points to the closer centroid as a cluster; (c) recalculating the positions of centroids of each cluster; (d), (e), and (f) iterations of assigning data points to clusters and recalculating the positions of centroids; (g) obtained results of k-means method.
